# Light-Regulated Gene Expression Patterns During Conidial Formation in *Aspergillus oryzae*

**DOI:** 10.3390/cimb47050373

**Published:** 2025-05-20

**Authors:** Shangfei Lin, Jiali Yang, Aixia Wang, Qiqi Fu, Shijie Huang, Muqing Liu

**Affiliations:** 1 College of Physics and Optoelectronic Engineering, Foshan University, Foshan 528231, China; 2 Institute of Future Lighting, Academy for Engineering and Technology, Fudan University, Shanghai 200433, China; sjhuang19@fudan.edu.cn (S.H.); mqliu@fudan.edu.cn (M.L.); 3 Department of Light Sources and Illuminating Engineering, School of Information Science and Technology, Fudan University, Shanghai 200433, China; jialiyang21@m.fudan.edu.cn (J.Y.); qqfu21@m.fudan.edu.cn (Q.F.); 4 Zhongshan-Fudan Joint Innovation Center, Zhongshan 528400, China

**Keywords:** photobiological effect, *Aspergillus oryzae*, light-regulated gene, conidial formation, *abaA* gene

## Abstract

With the effect of light on the conidial formation of *Aspergillus oryzae* now being known, the molecular mechanism of its light response has become a research hotspot. However, the light-regulated genes investigated in earlier studies do not clearly explain the light response patterns of related genes at the transcriptional level. This study employed RNA sequencing technology to preliminarily identify the light-regulated genes among the genes related to conidia production and photoreception in *A. oryzae* GDMCC 3.31. Subsequently, the effects of light dose on the light-regulated genes were analyzed by qRT-PCR. We identified a total of six genes (*tcsA*, *catA*, *gld1*, *Aowc-1*, *abaA*, and *AofphA*) as light-regulated genes. The expression pattern of *abaA* was dependent on the light spectrum and light dose. When the light dose was maintained at a high level, the *abaA* gene served as a red–green light-regulated gene. Otherwise, the *abaA* gene showed no response to light. The phytochrome-like gene *AofphA* was regulated by red and blue light with a biphasic response under varying light doses, suggesting the existence of a light dose threshold. These findings provide new targets for the photoresponse molecular mechanisms in *A. oryzae*.

## 1. Introduction

*Aspergillusoryzae* is an important industrial microorganism widely used in food fermentation and biotechnology [1,2,3,4]. Research has shown that light can cause significant changes in the phenotype of *A. oryzae* [5,6,7,8]. For example, the blue light spectrum has an obvious inhibitory effect on conidia production in *A. oryzae* GDMCC 3.31, while red light and green light have little impact [5]. These studies have also revelated effective optical parameters (light spectrum, light intensity, light mode, and light dose), among which the effect of the light spectrum is strongest. These optical parameters are appropriate targets for the study of the light response mechanism of spore formation.

If we look deep inside the cells of fungi, it can be seen that a major effect of photoreception is a global change in the transcriptome [9,10]. At present, more than 205 genes in the genome assembly of *A. oryzae* registered in the NCBI database have had their functions verified [11,12,13]. The central regulatory pathway composed of three transcriptional regulators (BrlA, AbaA, and WetA) plays a role in sequentially activating the development of conidial heads and controlling the expression of genes specific to asexual development [14,15]. The functions of these genes are almost conserved between *A. oryzae* and *Aspergillus nidulans* [16,17]. Any mutation in one of three genes causes asexual development to cease at a specific morphological stage. Furthermore, the normal formation of conidia also requires the assistance of signal proteins and developmental regulators, such as TcsA, VeA, LaeA, and FluG [18,19,20]. Therefore, when studying the light response mechanism of spore formation, the above genes were targeted.

A few researchers have carried out studies on these genes. Hatakeyama’s team demonstrated that the expressions of *brlA* and *veA* in the *A. oryzae* strain RIB40 remained unaffected under red light [21]. This result preliminarily ruled out the possibility of the regulation of *brlA* expression by the red light spectrum. However, they did not investigate the influences of the red light spectrum on other target genes nor did they consider the possible effect of other spectra on the *brlA* gene. Suzuki identified 67 white-light-regulated genes by comparing the RIB1187 and RIB40 strains using RNA sequencing technology [22]. The results suggested that even when the light spectrum is wide, light regulates many genes. Ruger identified the light-regulated conidiation genes in *A. nidulans* and determined the influence of light dose on the *brlA* mRNA levels [23]. They also did not further study the light response of other genes such as *abaA*. Although the above studies have clarified that some genes that respond to optical parameters, the efficiency of the research was low, so these results are insufficient for understanding the light response mechanism of spore formation. RNA sequencing technology, as a high-throughput transcriptome sequencing technology, may be an effective method to improve the efficiency of screening for genes that respond to light.

In addition to the genes related to conidia formation, since the photoreceptor is the starting point of the light response, its genes are also key. Unfortunately, no photoreceptor has been identified in *A. oryzae*; meanwhile, many photoreceptors have already been discovered in other filamentous fungi, and studies on the molecular mechanism of their light response have been carried out [24,25]. FphA was identified as the red light receptor in *A. nidulans* early 2005 [26,27]. It senses red and far-red light through photo-induced stable conformational conversion. Under red light, FphA represses the sexual development of *A. nidulans*. The results of genome-wide expression analysis showed that more than 500 genes in *A. nidulans* were regulated by light [9,23]. Moreover, the first blue light receptor in fungi, white collar 1 (WC-1), was identified in *Neurospora crassa,* and its mechanism has been explored [14,28]. These photoreceptors helped to find the potential photoreceptor genes in *A. oryzae* using methods such as the Basic Local Alignment Search Tool (BLAST), as well as to explore the light responses of photoreceptor-like genes.

Here, three light spectra were set up to irradiate *A. oryzae* GDMCC 3.31. We conducted RNA sequencing to explore the transcriptional changes in the genes related to spore formation or light sensing. Then, the light-regulated genes were screened. Subsequently, quantitative reverse-transcription PCR (qRT-PCR) was used to further assess how varying light doses influenced gene expression. This study provides new targets for the photoresponse molecular mechanisms in *A. oryzae*.

## 2. Materials and Methods

### 2.1. Fungal Species

*A. oryzae* strain GDMCC 3.31 was purchased from the Guangdong Microbial Culture Collection Center (GDMCC, Guangzhou, China). The samples were incubated on potato dextrose agar (PDA; CM123, Beijing Land Bridge Company, Beijing, China) plates at 28 °C for 5 d. The 3rd generation was obtained after two subcultures. Its spores were suspended in sterile water containing 0.002% (*v/v*) Tween 80 as well as 0.5% (*w/v*) NaCl and stored at 4 °C.

### 2.2. Culture Conditions

The PDA plates were prepared in advance, and each plate was covered with a small piece of cellophane. The spore suspension was diluted to 1 × 10^7^ CFU/mL as a conidial inoculum. Each plate was inoculated with 10 μL of diluted spore suspension and cultured in a light incubator (Spectracell-MU250L; Shanghai LightEngin Technology Co., Ltd., Shanghai, China) at 30 °C. All cultures were repeated in triplicate.

### 2.3. Light Conditions and Apparatus

To generate the RNA-sequencing samples, a high-dose light treatment was administered immediately following the inoculation of the spore suspension and continued for 72 h. The optical parameters for the high-light-dose treatments were established based on our previous studies [5,8]. We adopted the same light treatment protocols as in our prior research to facilitate a comparative analysis of the results. Given that the light spectrum exerted the most pronounced photobiological effects, it was prioritized as a key optical parameter. In this study, three different light spectra were incorporated into the high-light-dose treatment to assess their influence on the transcriptome (Table 1). All three light treatments were produced using LED light sources integrated into an incubator (Spectracell-MU250L; LightEngin Technology, Shanghai city, China), while darkness served as the control. Following irradiation, samples were collected and prepared for RNA sequencing, which allowed for the identification of the light-regulated genes among the target genes.

To further evaluate the effect of light dose on the screened genes, qRT-PCR samples were prepared. Considering that the growth of *A. oryzae* on the PDA medium containing cellophane was slower than that on the pure PDA medium and as *A. oryzae* remained in the stable phase when incubated for 40 to 72 h, we subjected *A. oryzae* in the stable phase to low-light-dose treatment. This approach mitigated the potential influence of growth status differences. Specifically, strains incubated in the dark for 40 h were exposed to a low-light dose for 30 min and then prepared for the low-light-dose treatment samples. Detailed parameters are provided in Table 1. For the other treatments, samples were prepared using the same method as for the RNA-sequencing samples. It should be noted that the light intensity and wavelength parameters for both light treatments were identical, with the only difference being the duration of light exposure. Darkness served as the control. Following the light treatments, the qRT-PCR technique was used to determine the relative gene expression.

### 2.4. RNA Extraction

Mycelia were harvested with a cell lifter and pulverized in liquid nitrogen. The total RNA was extracted from the mycelium with a TRIzol Reagent Kit (Invitrogen, Carlsbad, CA, USA) according to the manufacturer’s protocol. RNA quality was evaluated with an Agilent 2100 Bioanalyzer (Agilent Technologies Inc., Palo Alto, CA, USA).

### 2.5. Library Construction and Sequencing

Eukaryotic mRNA was enriched with Oligo(dT) beads (Thermo Fisher Scientific, Waltham, MA, USA). The enriched mRNA was broken up using a fragmentation buffer and reverse-transcribed into cDNA using random primers. The second-strand cDNA was synthesized with NEBNext Ultra RNA Library Prep Kit (NEB #7530, New England Biolabs, Ipswich, MA, USA). The cDNA fragments were purified using a QiaQuick PCR Extraction Kit (Qiagen, Venlo, The Netherlands), end-repaired, appended with poly(A), and ligated to Illumina sequencing adapters (Illumina, San Diego, CA, USA). The ligation products were sorted by size using agarose gel electrophoresis, amplified by PCR, and sequenced on an Illumina HiSeq2500 platform by Gene Denovo Biotechnology Co., Guangzhou, China.

### 2.6. Bioinformatics Analysis in RNA Sequencing

A reference genome index was constructed, and the clean paired-end reads were mapped to the reference genome of *A. oryzae* (assembly No. ASM18445v3) using HISAT2. The mapped reads from the sample were then assembled with StringTie (v. 1.3.1). All genes were annotated against the Gene Ontology (GO) database and the KEGG pathway database [29]. Genes that were not annotated in the reference genome had their gene IDs numbered by MSTRG.

All genes were analyzed, and gene expression levels were measured in terms of fragments per kilobase of exon model per million mapped reads (FPKM) values. Differential RNA expression analysis between treatment pairs was conducted using DESeq2. Genes with a false discovery rate (FDR) < 0.05 and an absolute fold change (|FC|) ≥ 2 were considered differentially expressed.

Among the results of the RNA sequencing, we searched for genes related to conidial formation and focused on the results. Moreover, by comparing the sequence of WC-1 and *A. oryzae* protein sequences, the FphA sequence, and *A. oryzae* protein sequences, we discovered two photoreceptor-like genes in *A. oryzae*, which we designated *Aowc-1* and *AofphA*. Detailed comparisons were conducted using the BLASTP tool with the National Center for Biotechnology Information (NCBI) database (https://blast.ncbi.nlm.nih.gov/Blast.cgi, accessed on 7 August 2024).

### 2.7. Relative Expression of Genes by qRT-PCR

Given that qRT-PCR offers higher sensitivity and specificity for individual genes, it was employed to assess the impact of high-light doses on gene expression and to validate the RNA-sequencing results. A total of twenty-four genes, primarily involved in sporulation and the mitogen-activated protein kinase (MAPK) signaling pathway, were analyzed using qRT-PCR.

To investigate the effect of a low-light dose on gene expression, qRT-PCR was performed on sixteen genes. Specific analyses were conducted using a real-time PCR system (QuantStudio 3; Thermo Fisher Scientific, Waltham, MA, USA). The *β-tubulin* gene served as the reference gene. The PCR conditions were as follows: 95 °C for 3 min, followed by 45 cycles of 15 s at 95 °C, and 30 s at 60 °C. All primers used are listed in Table S1.

### 2.8. Statistical Analyses of qRT-PCR

Each treatment was replicated at least three times. The relative expression was calculated using the 2^−ΔΔCt^ method (Table S2) [30]. Gene expression differences were analyzed with GraphPad Prism (v9.4.1) using a one-way ANOVA. All results are presented as average values. The symbols *, **, ***, and **** in the figures throughout this article represent statistical significance levels of *p* < 0.05, *p* < 0.01, *p* < 0.001, and *p* < 0.0001, respectively. Additionally, abbreviations Ctr, RL, GL, and BL represent the high-dose light treatments, improving clarity and conciseness. Similarly, abbreviations RL-30 min, GL-30 min, and BL-30 min are used to indicate low-dose light treatments. The correlation between the RNA sequencing and qRT-PCR was analyzed using a curve-fitting tool in Excel.

## 3. Results

### 3.1. Overall Regulation of Gene Expression by Light

**Figure 1:**
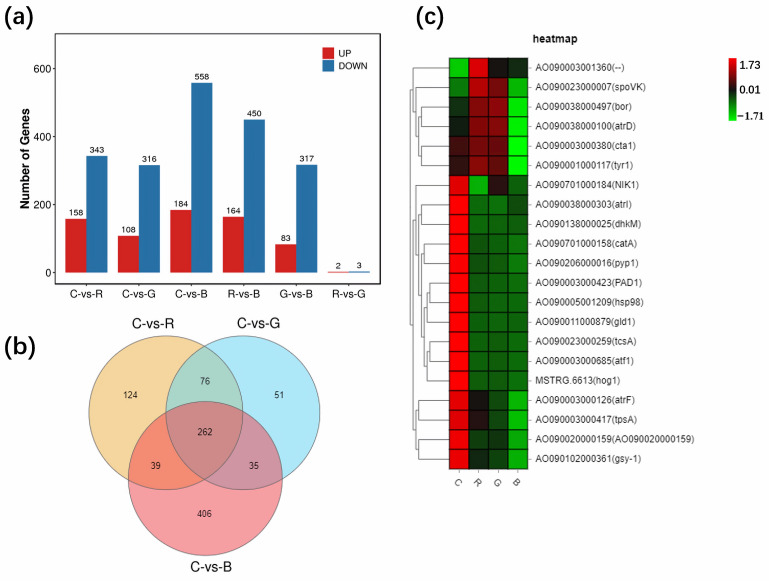
Plots of DEGs in treatment pairs. (**a**) Histogram of DEGs. The term C-vs-R refers to the comparison of the red light treatment and the dark control. The bars for C-vs-R represent the number of DEGs regulated by the red light, while the other terms and bars are labeled similarly. (**b**) Venn diagram of DEGs. (**c**) Heatmap of specific DEGs.

The overall results of RNA sequencing indicated that red light, green light, and blue light, respectively, regulated 501, 424, and 742 DEGs. Blue light had the most significant influence on *A. oryzae* GDMCC 3.31 (Figure 1a). Of these DEGs, 406 were selectively controlled by blue light (Figure 1b). Some products encoded by light-regulated genes were involved in asexual sporulation and the perception of light (Figure 1c).

### 3.2. Light-Regulated Genes Related to Conidial Formation

In the RNA-sequencing results, six regulator genes (*brlA*, *abaA*, *wetA*, *veA*, *laeA,* and *fluG*), mentioned in the Introduction Section, were similarly expressed between the light treatments and darkness. The detailed RNA-sequencing results are provided in Table S3. Furthermore, the qRT-PCR results confirmed five genes’ results (*brlA*, *wetA*, *veA*, *laeA,* and *fluG*), except for the *abaA* gene. The detailed qRT-PCR results are provided in Table S2. Consequently, it was concluded that these five regulator genes do no respond to the light spectrum at the transcriptional level.

**Figure 2:**
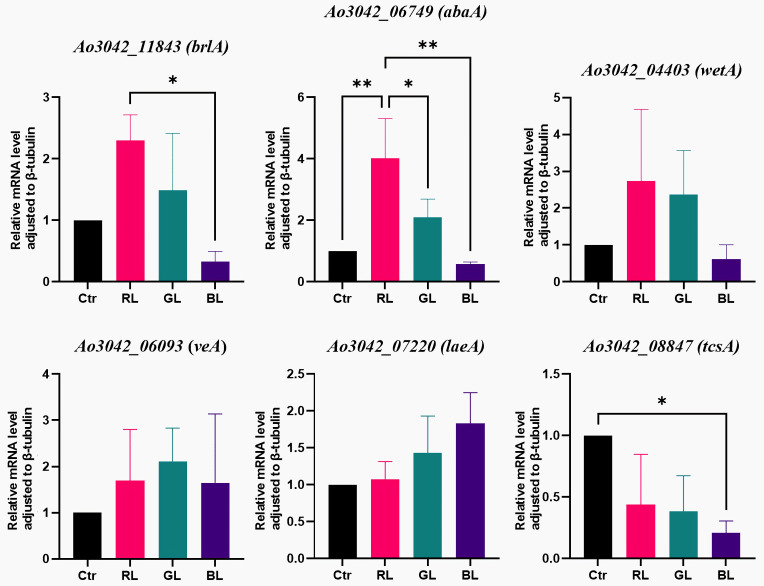
The qRT-PCR results of genes related to conidial formation. Ctr, RL, GL, and BL represented the control, red light, green light, and blue light treatments, respectively. Each gene has its gene tags numbered as Ao3042_XXXXX from the *A. oryzae* 3.042 genome from the NCBI database. Detail results are recorded in Table S2. The symbols * and ** represented statistical significance levels of *p* < 0.05 and *p* < 0.01, respectively.

The qRT-PCR results indicated that the expression of the *abaA* gene was similar between two light treatments (green light and blue light) and darkness. However, *abaA* was significantly upregulated by red light (*p* = 0.0038, Figure 2, Table S2). The mRNA level of the *abaA* gene under the red light treatment was 4.02-fold higher than that in darkness. Moreover, significant differences were found between the red light treatments and the other two light treatments. This inconsistency in results was expected and mainly stemmed from the differences between the two detection methods. The FPKM levels of the *abaA* gene under the light treatments and the control group ranged from 24 to 78, which are low expression levels in RNA sequencing (Table S3). These low FPKM levels may have limited the ability to detect differential expressions. Given the higher sensitivity and specificity of qRT-PCR, especially for detecting genes with low expression levels, expression differences were more easily identified using qRT-PCR. Therefore, when the light dose was maintained at a high level, the *abaA* gene was considered a red-light-regulated gene.

**Figure 3:**
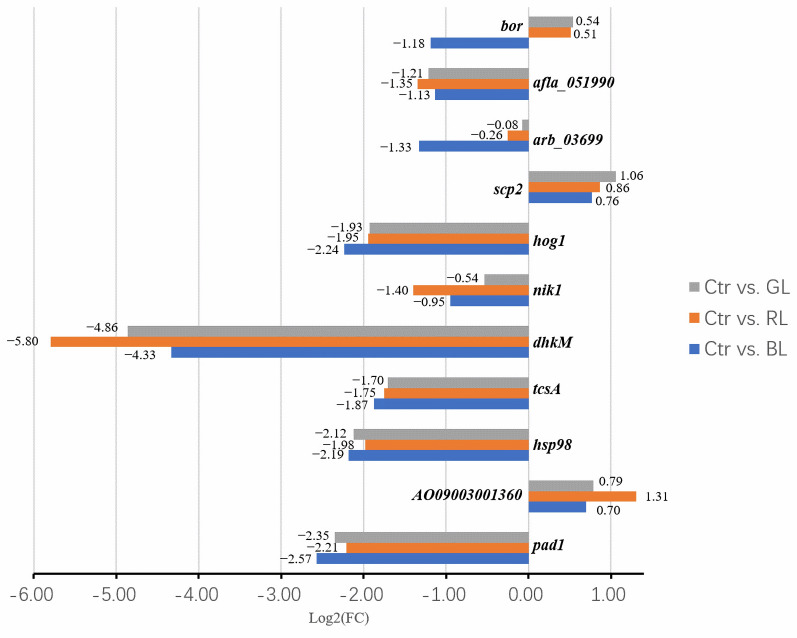
The RNA-sequencing results of DEGs related to asexual sporulation. Genes with FDR < 0.05 and Log2 (FC) ≥ 1 were considered differentially expressed between light treatments and darkness. The positive and negative signs on the horizontal coordinates represent positive and negative regulation, respectively. Ctr, RL, GL, and BL represent the control, red light, green light, and blue light treatments, respectively. Other genes without differences in expression are also recorded in Table S3.

Furthermore, we delved deeper into two GO processes, namely, asexual sporulation (GO: 0030436) and sporulation, resulting in the development of a cellular spore (GO: 0030435), and we found a total of eleven DEGs. The corresponding RNA-sequencing results are recorded in Table S3. As illustrated in Figure 3, six DEGs (*afla_051990*, *hog1*, *dhkM*, *tcsA*, *hsp98*, and *pad1*) were downregulated under all three light spectra. The most strongly inhibited gene was *dhkM*, which, along with three other DEGs (*hsp98*, *pad1*, and *tcsA*), is involved in signal transduction, stress response, and resistance in *A. oryzae*. The expression of *dhkM* was the most strongly suppressed by red light, with its expression under darkness being 55.2 times higher than under red light. However, the function of *dhkM* remains uncharacterized in *A. oryzae* to date. Meanwhile, *tcsA* was predicted to encode a sensor histidine kinase in *A. oryzae*, part of an osmotic two-component system that regulates DNA-templated transcription. The RNA-sequencing results showed that the expression of the *tcsA* gene was downregulated by all three light spectra (Figure 3). The FPKM level of *tcsA* ranged from 23 to 85, which are considered low expression levels. The FPKM ratio of the blue light treatment to darkness was 0.27. Furthermore, the qRT-PCR results only corroborated the results under the blue light treatment (Figure 2). The expression of *tcsA* was significantly downregulated by blue light, while the expressions under the other two light treatments showed no significant differences compared to darkness (Figure 2 and Table S2). The mRNA level of *tcsA* under blue light treatment was 0.21 times lower than in darkness (*p* = 0.0219). Based on the differences between the two detection methods and using the same analysis strategy as for the *abaA* gene, with the light dose maintained at a high level, the *tcsA* gene was considered a blue-light-regulated gene.

### 3.3. Light-Regulated Genes Among the Potential Photoreceptor-like Genes

**Figure 4:**
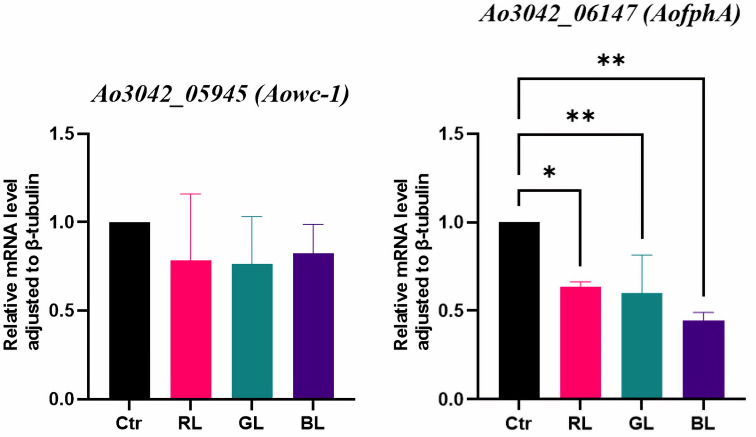
The qRT-PCR results of photoreceptor-like genes. Ao3042_05945 and Ao3042_06147 are the gene tags of *Aowc-1* and *AofphA*, respectively. The gene tags were from the *A. oryzae* 3.042 genome of NCBI database. Ctr, RL, GL, and BL represent the dark, red light, green light, and blue light treatments, respectively. The symbols * and ** represented statistical significance levels of *p* < 0.05 and *p* < 0.01, respectively.

Regarding photoreceptor-like genes, we found two genes (*Aowc-1* and *AofphA*) of *A. oryzae* with the potential to be photoreceptor genes using BLAST tools. The sequence of *Aowc-1* is similar to *wc-1* of *N. crassa*, which encodes the blue light receptor WC-1. WC-1 is a transcription factor in *N. crassa* that initiates the light response and generates numerous light-responsive proteins [31]. The sequence of *AofphA* is similar to *fphA* of *A. nidulans*, which encodes the phytochrome FphA. FphA represses sexual development under red light [26]. The RNA-sequencing results showed no significant differences in the expressions of these two genes under the light treatments and the dark control (Table S3). Furthermore, the qRT-PCR results corroborated the findings for *Aowc-1* (Figure 4). The qRT-PCR results indicated that the expression of *AofphA* was significantly suppressed by all three light spectra (Figure 4), with blue light exhibiting the most significant suppression. The mRNA level of *AofphA* under the blue light treatment was only 0.45 times that in the dark (*p*-value = 0.0012, Table S2). With the light dose maintained at a high level, the *Aowc-1* gene had no response to the light spectrum at the transcriptional level, and the *AofphA* gene was found to be a light-regulated gene.

### 3.4. Results of Genes in the MAPK Pathway

Studies have reported that light signals activate the MAPK signaling pathway [32,33]. As such, the DEGs involved in this pathway were of interest. During the RNA-sequencing analysis, two genes, *AO090020000466* and *AO090701000642*, were predicted as MAP kinase genes from the KEGG database. Additionally, a new gene (numbered *MSTRG.6613*) was identified in *A. oryzae* GDMCC 3.31. This gene was also predicted to encode MAP kinase, as its sequence has very high identity with the MAP kinase’s complete CDS sequence (GenBank No. BAD89083.1).

**Figure 5:**
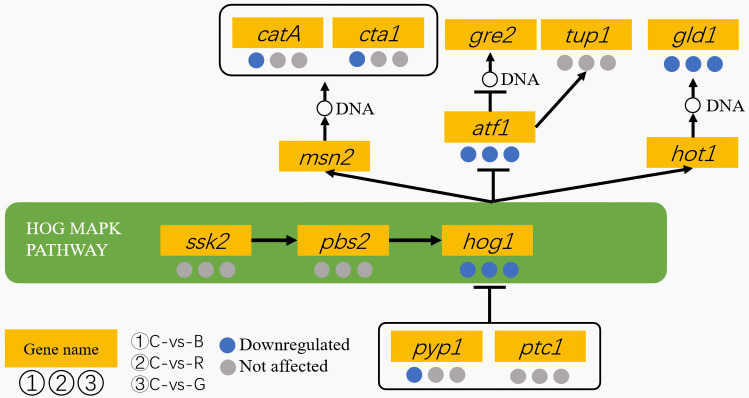
RNA-sequencing results of genes in the central and downstream HOG MAPK pathway. The circled numbers 1, 2, and 3 represented the results of blue, red, and green light treatments, respectively. The results are displayed in color, with red, blue, and gray indicating upregulation, downregulation, and no effect, respectively. Since three genes (*msn2*, *gre2* and *hot1*) were not identified, the corresponding results are not presented. Detailed FPKM levels are provided in Table S4.

**Figure 6:**
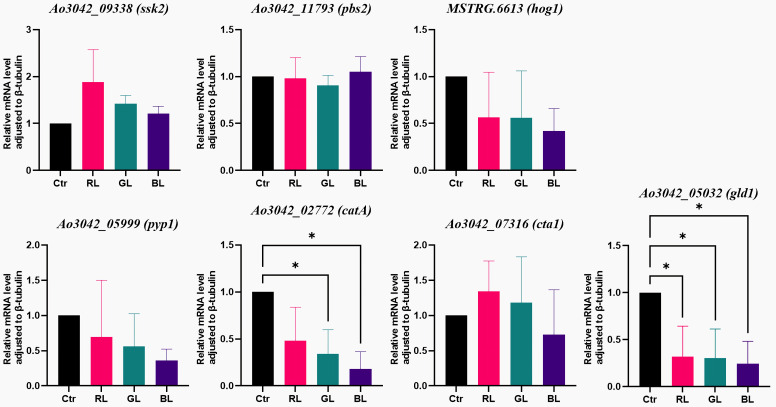
The qRT-PCR results of genes involved in HOG MAPK pathway. These genes were differentially expressed under different light spectrum treatments or darkness. The symbol * represented statistical significance levels of *p* < 0.05.

The RNA sequencing results for the genes in the high-osmolarity (HOG) MAPK pathway are recorded in Table S4. The RNA-sequencing results revealed that a total of six DEGs (*MSTRG.6613*, *atf1*, *pyp1*, *gld1*, *cta1*, and *catA*) exhibited differential expression under light treatments and darkness (Figure 5). In detail, the expressions of the *ssk2*, *pbs2*, *AO090020000466* and *AO090701000642* genes, which encode the key kinases, were similar under the light treatments and darkness. Their FPKM levels were low in all groups. The subsequent qRT-PCR results confirmed these findings (Figure 6 and Table S2). Additionally, the expression of *MSTRG.6613* was obviously different between the light treatments and the dark control (Table S4). However, further qRT-PCR analysis showed the differences were not significant (Figure 6, Table S2). Given the uncharacterized biological function of *MSTRG.6613* and the inconsistent results from the two detection methods, combined with the results for the other genes, it was concluded that the MAP kinase genes are not light-regulated.

Regarding downstream genes, the RNA-sequencing results showed that four stress-protection-related genes (*catA*, *cta1*, *atf1*, and *gld1*) responded to light (Figure 5). Both the *catA* and *cta1* genes were predicted to encode catalases, which protect cells from the toxic effects of hydrogen peroxide. The RNA-sequencing results indicated that the expressions of *catA* and *cta1* under blue light were obviously lower than in the dark (Table S4). The FPKM levels of *catA* under the light treatments were low. Additionally, the qRT-PCR results did not support the findings for the *cta1* gene, but they revealed that the *catA* gene was significantly suppressed by both green light and blue light (*p*-value = 0.0383 and 0.0126, Figure 6 and Table S2). The RNA-sequencing results also revealed that the *gld1* gene, which encodes the enzyme glycerol-3-phosphate dehydrogenase, was downregulated by all three types of light, with the greatest reduction occurring under blue light (Figure 5 and Table S4). These findings were further validated by qRT-PCR (Figure 6 and Table S2). The mRNA level of *gld1* under blue light was only 0.24 times that in the dark (*p*-value = 0.0266, Figure 6). In summary, with the light dose maintained at a high level, the *catA* gene was found to be a blue- and green-light-regulated gene, and the *gld1* gene was found to be a light-regulated gene.

### 3.5. Light-Regulated Genes Were Sensitive to Light Dose

In the above studies, light-regulated genes were screened based on the light spectrum, with the light dose parameter maintained at a high level. Here, we further explored the sensitivity of these genes to different light doses.

**Figure 7:**
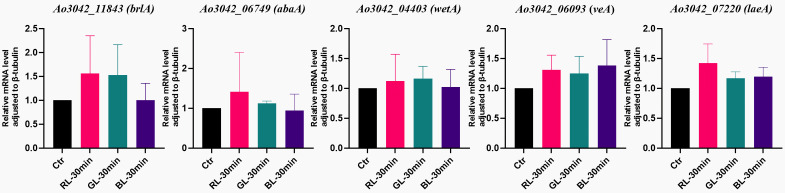
Expression of sporulation-related genes under light at a low dose. Ctr, RL, GL, and BL represent the control, red light, green light, and blue light treatments, respectively.

**Figure 8:**
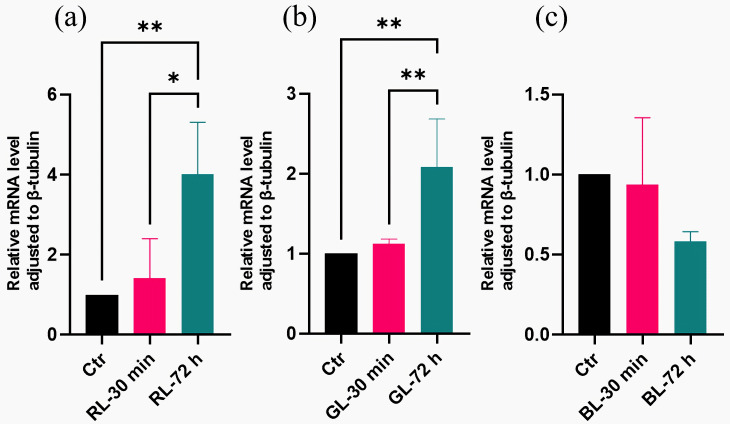
Expression of *abaA* gene under different light doses. Subfigures (**a**–**c**) are the mRNA level results of *abaA* when the spectrum involved red light, green light, and blue light, respectively. The symbols * and ** represented statistical significance levels of *p* < 0.05 and *p* < 0.01, respectively.

Regarding the genes related to conidia formation, the qRT-PCR results showed that the expressions of certain genes (*brlA*, *wetA*, *veA*, and *laeA*) under low-light-dose treatments were comparable to those in the dark (Figure 7). These findings were consistent with those observed under high-light doses (Figure 2). Meanwhile, the mRNA levels of *abaA* under low-light-dose treatments were similar to those in the dark, regardless of the light spectrum (Figure 7). These results differed from the regulation observed under high light doses, suggesting a distinct expression pattern (Figure 2). two-way ANOVA revealed a significant interaction between the light spectrum and light dose parameters (*p*-value = 0.0056). The unique regulation of *abaA* by light dose is shown in Figure 8, suggesting a specific light dose threshold between 30 min and 72 h. Therefore, four genes (*brlA*, *wetA*, *veA*, and *laeA*) related to conidial formation showed no sensitivity to the two light parameters (light spectrum and light dose). The expression pattern of the *abaA* gene was dependent on both the light spectrum and light dose. When the light dose was maintained at a high level, the *abaA* gene served as a red- and green-light-regulated gene. Otherwise, the *abaA* gene did not respond to light.

**Figure 9:**
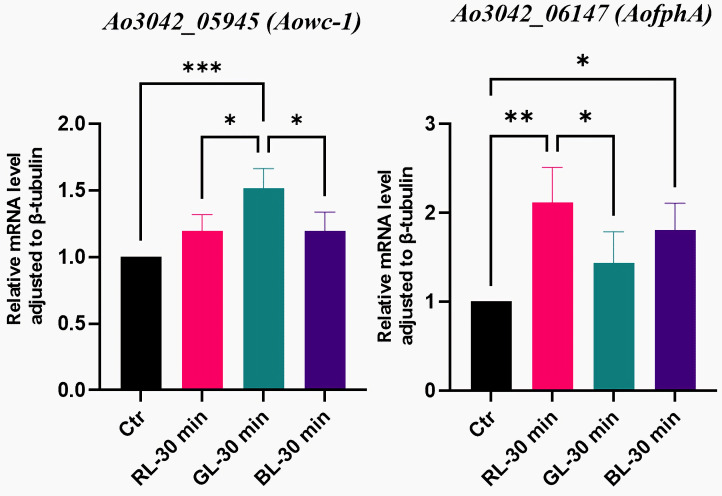
Expression of photoreceptor-like genes under low-dose light treatments. The symbols *, **, and *** represented statistical significance levels of *p* < 0.05, *p* < 0.01, and *p* < 0.001, respectively.

Regarding photoreceptor-like genes, two genes responded to light under a low dose (Figure 9). The mRNA levels of *Aowc-1* were unaffected by red or blue light at low doses, consistent with the results observed at high doses. This suggested that red and blue light did not influence the expression of *Aowc-1*, regardless of the light dose. In contrast, *Aowc-1* was significantly induced by green light (*p*-value = 0.0002, Figure 9). Its mRNA level under low doses of green light were 1.52 times higher than those in the dark. An additional two-way ANOVA revealed a significant main effect of light dose (*p*-value = 0.0038). These findings suggested that the light regulation of the *Aowc-1* gene was dependent on the light dose. Specifically, *Aowc-1* gene was found to be a green-light-regulated gene only at low doses.

The qRT-PCR results demonstrated that the expression of *AofphA* was significantly stimulated by both blue and red light at low doses, whereas green light had minimal effects (Figure 9). Notably, the strongest induction occurred under red light, with an mRNA level 2.12 times larger than that in darkness (*p*-value = 0.0026). This finding starkly contrasted the results for high doses of red and blue light (Figure 4). Additionally, a two-way ANOVA suggested no interaction effects between the light dose and spectrum, while indicating a significant main effect attributed to light dose. Collectively, these findings illustrated that *AofphA* exhibited a biphasic response to light, with its response contingent upon a specific light dose threshold.

## 4. Discussion

The RNA-sequencing results indicated that after 72 hours of continuous irradiation, numerous gene expressions were altered, with blue light exhibiting the most significant effects. Different light spectra suppressed different gene expressions, suggesting that the organism possesses a complex light-sensing mechanism capable of distinguishing between different wavelengths and eliciting the corresponding physiological responses.

To identify light-regulated genes, we analyzed some target genes involved in light perception, conidia production, and the HOG MAPK signaling pathway. Through our analysis of RNA-sequencing data and qRT-PCR results, we screened six light-regulated genes, including *abaA* and *catA*. Notably, we found that the expression pattern of *abaA*, which encodes the central regulator of conidiation, was dependent on the light spectrum and light dose. When the light dose was maintained at a high level, the *abaA* gene functioned as a red- and green-light-regulated gene. The factor AbaA contains an ATTS DNA-binding domain and, like BrlA, is essential for the transcriptional activation of conidiation-specific genes. Typically, AbaA induces the expression of *wetA*, subsequently stimulating the expression of additional conidiophore-specific genes. The results for *brlA*, one of the key genes, under the various light conditions were consistent with previous results on *A. oryzae* [21,34]. It is well known that these three factors control gene expression at specific times and locations during the development of conidiophores and conidia maturation, functioning as regulatory switches. The aberrant expression of any of these genes can disrupt normal growth and conidiation of the strain [35,36,37,38]. Previous studies demonstrated that three types of light did not destroy the conidiation or vegetative growth of *A. oryzae* but instead played a regulatory role [5,21,34,39]. Thus, it is evident that all three genes (*brlA*, *abaA*, and *wetA*) are expressed normally and perform their biological functions under light conditions. Our transcriptional level results provide supporting evidence for these conclusions.

The induction of *abaA* by red and green light at high doses is a novel finding in the study of the light response of *A. oryzae*. This observed trend is inconsistent with the lack of trend for red light in a previous study. A previous study indicated that red light did not affect the number of conidia produced [5]. Based on the following two points, we believe that the difference in the trend between these two studies is normal. On the one hand, the results derived from two different levels of analysis should not be directly compared. The spore count reflects the accumulation of the final product and does not capture the dynamic fluctuations occurring during the process. In our study, the mRNA levels of *abaA* were measured immediately after light treatment and therefore represent the transcription of the *A. oryzae* genome at that specific time point. However, these transcription products must undergo multiple biological processes, such as translation, before the conidia are finally formed. Therefore, these two results cannot be compared. On the other hand, the result for the *wetA* gene offers a tip. The formation of conidia is regulated by the BrlA–AbaA–WetA pathway, where BrlA activates the expression of *abaA*, which in turn activates the expression of *wetA*. Our results showed that although red and green light upregulated *abaA*, the expression of its downstream *wetA* gene was unaffected. This pathway has strict temporal regulatory characteristics. An isolated increase in the mRNA level of *abaA* may not reach the threshold to trigger the expression of *wetA*, potentially resulting in the failure to activate the structural genes related to spore formation. Consequently, this could lead to a lack of significant changes in spore yield.

Regarding the results for *Aowc-1* and *AofphA*, our findings indicate for the first time that under specific light conditions, photoreceptor-like genes respond to light at the transcriptional level. *AofphA* in *A. oryzae* exhibited a biphasic response correlated with light dose thresholds. Low-light doses of red and blue light induced its expression, while when the light dose increased, the three types of light inhibited its expression. Moreover, this biphasic effect of light-regulated genes is not unique to *A. oryzae*, as similar phenomena have been observed in other fungal species [40,41,42]. In the absence of evidence at the protein levels, the underlying mechanisms of this effect remain challenging to elucidate. We propose that this response represents an evolutionary adaptation that enables *A. oryzae* to balance resource utilization and survival strategies under varying light doses. In the future, we will perform the biological functional characterization of *AofphA* and further explore its response to light at the protein level.

## 5. Conclusions

In conclusion, our study focused on light-regulated genes among those related to conidial formation and light sensing in *A. oryzae* GDMCC 3.31. The results indicated that four conserved regulator genes (*brlA*, *wetA*, *veA*, and *laeA*) related to spore formation did not respond to light spectra or light doses. Meanwhile, the expression pattern of *abaA* was dependent on the light spectrum and light dose. When the light dose was maintained at a high level, the *abaA* gene was red- and green-light-regulated. Otherwise, the *abaA* gene did not respond to light. Finally, we screened a total of six light-regulated genes (*tcsA*, *catA*, *gld1*, *Aowc-1*, *abaA*, and *AofphA*). When the light dose was maintained at a high level, the *tcsA*, *catA*, and *gld1* genes were blue light-regulated, a blue-green light-regulated gene, and a light-regulated gene, respectively. When the light dose was decreased to a low level, *Aowc-1* was found to be a green light-regulated gene. The expression pattern of the *AofphA* gene showed a biphasic response to light, with its response contingent upon a specific light dose threshold. These results clearly revealed the expression patterns of *A. oryzae* genes under light exposure, deepening the understanding of the photoresponse mechanism of *A. oryzae*.

## Figures and Tables

**Table 1:** Parameters of light treatments.

Parameter	High Light Dose Treatment			Low Light Dose Treatment		
Light source	Blue	Green	Red	Blue	Green	Red
Light wavelength (nm)	475	520	630	475	520	630
Full width at half maximum	25	37	21	25	37	21
Average irradiance (W·m^–2^)	19.87	17.87	15.63	19.87	17.87	15.63
Irradiated time	72 h			30 min		
Light dose (kJ·m^–2^)	5150	4632	4051	35.8	32.2	28.1
Light intensity (μmol photon m^−2^ s^−1^)	80					

## Data Availability

Raw data were uploaded to the SRA library (accession number: PRJNA909033, Table S5).

## Supplementary Materials

The following supporting information can be downloaded at: https://www.mdpi.com/article/10.3390/cimb47050373/s1.
